# Racial-Ethnic Residential Segregation and Sleep Health among US Adults: Associations by Race and Ethnicity, Sex/Gender, and Neighborhood-Level Poverty

**DOI:** 10.1007/s40615-025-02322-y

**Published:** 2025-03-31

**Authors:** Symielle A. Gaston, Jesse Wilkerson, Nathaniel MacNell, W. Braxton Jackson II, Lu Dong, Chandra L. Jackson

**Affiliations:** 1 Epidemiology Branch, National Institute of Environmental Health Sciences, National Institutes of Health, 111 TW Alexander Drive, Research Triangle Park, NC 27709 USA; 2 DLH, LLC, Bethesda, MD USA; 3 RAND Corporation, Santa Monica, CA USA; 4 Division of Intramural Research, National Institute on Minority Health and Health Disparities, National Institutes of Health, Bethesda, MD USA

**Keywords:** Neighborhood environment, Systemic racism, Poverty, Sleep duration, Sleep quality, Racial groups

## Abstract

**Introduction:**

Although racial-ethnic residential segregation (RRS) is hypothesized to contribute to sleep disparities by concentrating poverty and impairing sleep among minoritized racial-ethnic groups, feelings of belonging within relatively homogenous neighborhoods may be protective against poor sleep. Yet, empirical studies are sparse.

**Methods:**

To investigate RRS–sleep health associations and determine potential modifiers among US adults, we linked National Health Interview Survey data (2011–2017) to 2012 and 2017 American Community Survey census tract-level data. We used the local Getis-Ord G_i_* statistic to categorize RRS (high, medium, low [reference]). Using survey-weighted, Poisson regression with robust variance, we estimated prevalence ratios (PRs) and 95% confidence intervals (CIs) for self-reported sleep health measures. We also performed Wald tests for interactions by race-ethnicity, sex/gender, race-ethnicity-by-sex/gender intersectional category, and neighborhood-level poverty.

**Results:**

Among 126,539 participants (mean age ± SE = 46 ± 0.1 years), high RRS was most common among non-Hispanic (NH)-Black (38%), followed by NH-Asian and non-Mexican Latine (34%), Mexican Latine (30%), and NH-White adults (17%). Across races-ethnicities and sexes/genders (both p-interaction > 0.05), high vs. low RRS was associated with a 6% lower prevalence of short sleep duration (< 7-h: PR = 0.94 [95% CI:0.91–0.97]), an 11% lower prevalence of long sleep duration (> 9-h: PR = 0.89 [0.80–0.99]), and a 2% higher prevalence of restorative sleep (PR = 1.02 [1.01–1.04]). Associations with a lower prevalence of trouble falling asleep were stronger among men vs. women. Race-ethnicity-by-sex/gender group membership and neighborhood-level poverty modified associations with sleep duration and quality without consistent patterns.

**Conclusion:**

RRS was associated with more favorable sleep health among US adults with variation by key modifiers (e.g., sex). Strategies that leverage potentially protective social factors while promoting equitable resources across diverse neighborhoods may help address sleep health disparities.

**Supplementary Information:**

The online version contains supplementary material available at 10.1007/s40615-025-02322-y.

## Introduction

Sleep insufficiency, or obtaining less than the recommended amount of sleep, and sleep disorders are public health problems, both globally and in the United States (US) [1, 2]. Among US adults, approximately one in three do not obtain the recommended sleep duration of at least seven hours per night [3, 4]. Further, sleep health disparities exist among socially relegated groups [4–8]. For instance, minoritized racial and ethnic groups generally have worse sleep across multiple dimensions, and women report higher burdens of insomnia symptoms compared to men [5, 6, 8]. Given the substantial role of sleep health in contributing to overall health and disparities in health outcomes, sleep health disparities are of public health significance [8]. Thus, optimizing sleep health for all is essential, and elucidating exposures that impact sleep is of paramount public health importance. Extant literature supports that sleep health is influenced by physical and social environments [9, 10]; therefore, environmental factors, which are modifiable, may prove effective as sleep intervention targets.

Structural racism, or “the totality of ways in which societies foster racial discrimination through mutually reinforcing systems” that then reinforce discriminatory culture and practices, is a hypothesized fundamental environmental contributor to health disparities [11]; however, the pathways through which it might contribute to sleep health disparities require further study [12]. As one component of structural racism that operates through the persistent patterning of neighborhood environments, racial and ethnic residential segregation (RRS) is hypothesized to promote health disparities by isolating minoritized racial and ethnic groups in areas of higher poverty/concentrated disadvantage with higher exposure to environmental hazards (e.g., pollution; crime leading to less safety) and limited access to health promoting resources (e.g., grocery stores; greenspace) [11, 13, 14]. Consequently, RRS has been associated with myriad poor health outcomes (e.g., obesity, hypertension) that are impacted by sleep and that are more prevalent among minoritized racial and ethnic groups [15–20].

Conversely, some prior literature supports potentially protective effects of RRS. For instance, RRS in the form of ethnic enclaves may provide health benefits through preserving cultural and social connectedness, particularly among newer immigrant populations [16]. Moreover, prior studies reported protection against discrimination and increased physical activity among racially and ethnically minoritized individuals in highly segregated neighborhoods [16, 19, 21, 22]. Further, the relationship between RRS and health may vary by the health outcome under study and by racial-ethnic group [19, 23]. For instance, a recent study found (1) no association between RRS and physical nor mental health among White adults; (2) worse physical health among Black adults in more segregated neighborhoods; and (3) conversely, better mental health among Black adults in more segregated neighborhoods [23].

Hypothesized pathways explaining the associations are related to feelings of belongingness that may promote better health behaviors and mental health among racially and ethnically minoritized people in more segregated neighborhoods [22, 23]. However, regarding sleep, epidemiologists generally theorize that RRS causes sleep disparities with potential explanations including the disproportionate isolation or concentration of minoritized racial and ethnic groups in neighborhoods characterized by low socioeconomic status (SES) and environmental hazards (e.g., noise, air pollutants), each of which are associated with poor sleep health [8–10, 24–26]. Nonetheless, empirical data are sparse.

The limited studies that included sleep data suggest adverse relationships between RRS and both short sleep duration and adherence to continuous positive airway pressure (CPAP) therapy [27–29]. However, these studies lacked data on sleep quality dimensions. Moreover, sleep disparities have been found to vary by race and ethnicity and gender – among other social categories [4–8]; therefore, varying magnitudes of sleep differences across social groups are possible. In fact, the impact of intersectionality (i.e., differences that result from varying levels of power and privilege [or lack thereof] related to social group memberships) [30, 31] on sleep health has been demonstrated among minoritized racial and ethnic groups with membership in other marginalized groups (e.g., sexual and gender minoritized, women) [6, 32]. Based on the intersectionality framework for public health posited by Lisa Bowleg as inspired by scholars like Kimberle Crenshaw, hypothesized pathways occur through negative experiences and stressors related to intersections [30, 31, 33, 34] – for instance, between racism and sexism – compounding to negatively influence sleep health [5]. Thus, investigations of intersectionality in the RRS-sleep relationship are warranted. Lastly, given the conflicting literature suggesting both positive and negative consequences of RRS on health and the hypothesized pathways through economic disadvantage [13, 15–22], it is important to consider how associations between RRS and sleep may vary by neighborhood-level socioeconomic status.

To address these important research gaps, we sought to advance the literature by conducting the first study to investigate associations between RRS and multiple dimensions of sleep among a nationally representative sample of Asian, Black, Latine, and White adults in the US. Secondly, we sought to determine whether these RRS-sleep associations varied by race and ethnicity, sex/gender, the intersection of race and ethnicity with sex/gender, and neighborhood-level poverty. Although prior literature on the direction of associations between RRS and health behaviors is mixed, most studies suggest RRS as an adverse determinant of health [15, 17, 19, 22, 23]. Therefore, we hypothesized that (1) higher RRS is associated with worse sleep health, overall; (2) RRS-sleep associations are stronger among women and members of multiple marginalized groups (e.g., Black women); and (3) neighborhood-level poverty modifies associations. Specifically, upon stratification by neighborhood-level poverty, RRS will be associated with poorer sleep in high-poverty neighborhoods and with better sleep in low-poverty neighborhoods due to the absence of concentrated disadvantage and increased feelings of belonging in more racially and ethnically homogenous neighborhoods (i.e., ethnic enclaves) [22, 23].

## Methods

### Data Source: National Health Interview Survey

The most recently publicly available data at the time of our analysis were pooled from the annually administered National Health Interview Survey (NHIS) for the years 2011 to 2017 (response rate = 59% [range: 53% (2017)—66% (2011)]) by using Integrated Public Use Microdata Series (IPUMS) NHIS [35]. NHIS geographic data is restricted-use and was only available for analysis through access to the National Center for Health Statistics Restricted Data Center (RDC). Details on NHIS sampling, recruitment, and administration are provided elsewhere [36]. Briefly, after multistage sampling, face-to-face computer assisted interviews were administered by trained personnel in order to collect health data from individuals in sampled households to represent the non-institutionalized civilian US population. All participants provided informed consent. Data collection for the NHIS and analysis of restricted-use NHIS data were approved by the National Center for Health Statistics Ethics Review Board. Approval for use of de-identified, secondary, publicly available NHIS data is exempt from the federal regulations for the protection of human research participants and, therefore, was waived by the National Institutes of Health Institutional Review Board.

### Study Population

In the NHIS from 2011 to 2017, 232,235 sampled adults aged 18 years and older were surveyed. Following the 2016 US Office of Management and Budget standards of reporting race and ethnicity [37], NHIS interviewers collected self-identified race and ethnicity from participants. Participants reported whether they considered themselves Hispanic or Latino (hereafter, Latine used as a gender neutral term) and could select multiple categories that best described their race. All participants who identified as non-Hispanic Asian (hereafter Asian), non-Hispanic Black or African American (hereafter Black), Mexican Latine of any race, non-Mexican Latine of any race, or non-Hispanic White (hereafter White) were eligible for the current study (*n* = 225,718). We considered Mexican Latine and non-Mexican Latine participants separately due to prior evidence of intragroup differences in sleep among Latine adults [38], the NHIS data collection that allowed for the distinction between groups, and because this dichotomization resulted in a sufficient sample size in each stratum. Participants of other racial and ethnic groups (i.e., American Indian/Alaska Native, Native Hawaiian/Pacific Islander, multiracial, or a racial and ethnic group other than the aforementioned groups) were ineligible due to small sample sizes and heterogeneity if groups were combined. After exclusion criteria (i.e., currently pregnant; diagnosis of cancer, stroke, or heart disease; missing data for covariates included in models) were applied to publicly available data, 155,697 participants remained (Supplemental Table 1). After further exclusions related to restricted use data (e.g., unsuccessful geographical data linkage to external data) were applied at the RDC, the final analytic sample comprised 126,539 adults. 

**Table 1:** Age-standardized sociodemographic, health behavior, and clinical characteristics among Asian, Black, Hispanic/Latine, and White Adults, National Health Interview Survey, 2011–2017 (*N* = 126,539) ^a^

	Overall			Asian (non-Hispanic)			Black (non-Hispanic)		
	All (*N* = 126,539)	Men (*n* = 59,253)	Women (*n* = 67,286)	All (*N* = 6,156)	Men (*n* = 3,063)	Women (*n* = 3,093)	All (*N* = 18,411)	Men (*n* = 7,396)	Women (*n* = 11,015)
Sociodemographic characteristics									
Age, years—mean (SE)	45.9 (0.1)	44.8 (0.1)	46.8 (0.1)	42.7 (0.3)	41.9 (0.4)	43.5 (0.4)	44.4 (0.2)	44.0 (0.3)	44.7 (0.3)
Living in poverty (< 100% Federal Poverty Level)	14.6	12.7	16.4	13.6	12.9	14.3	25.7	20.4	29.6
Annual household income									
< $35,000	36.6	33.0	39.9	29.8	28.4	31.2	52.9	46.5	57.4
$35,000-$74,999	30.9	31.9	30.1	27.2	27.5	26.9	29.2	31.6	27.5
≥ $75,000	32.5	35.1	30.1	43.0	44.0	41.9	18.0	21.9	15.1
Educational attainment									
< High school	10.9	11.7	10.3	7.3	6.8	7.7	15.0	14.6	15.2
High school graduate	25.0	26.2	24.0	14.1	12.6	15.6	28.2	31.4	25.9
Some college	19.9	19.2	20.5	13.3	13.4	13.2	23.3	22.4	23.9
≥ College	44.1	43.0	45.2	65.4	67.2	63.6	33.6	31.6	35.0
Unemployed/not in the labor force	32.5	26.8	37.6	26.3	19.6	33.1	35.4	32.4	37.5
Occupational category									
Professional/management	20.4	24.2	17.0	35.0	44.2	25.8	13.1	13.8	12.6
Support services	45.5	27.2	62.2	43.4	30.8	56.0	47.5	28.2	61.3
Laborers	34.1	48.7	20.9	21.6	25.1	18.2	39.4	58.0	26.2
US-born (yes)	85.4	84.3	86.5	23.1	23.0	23.3	87.9	85.3	89.7
Region of residence									
Northeast	19.9	19.8	20.1	27.9	28.9	27.0	15.8	15.4	16.0
Midwest	29.4	30.1	28.8	21.1	21.5	20.8	20.5	21.5	19.9
South	41.6	40.8	42.3	34.5	34.8	34.3	61.8	60.7	62.5
West	9.1	9.4	8.7	16.4	14.9	18.0	1.9	2.4	1.6
Housing arrangement									
Government-assisted renter	4.1	2.5	5.5	2.3	1.8	2.7	12.0	7.1	15.5
Unassisted renter	34.8	36.5	33.2	42.6	47.0	38.2	47.4	50.5	45.1
Homeowner	61.2	61.0	61.3	55.1	51.2	59.1	40.6	42.3	39.4
Housing type									
Apartment/house	95.4	95.2	95.7	99.1	99.2	99.0	96.9	96.6	97.1
Mobile home/trailer	4.6	4.8	4.3	0.9	0.8	1.0	3.1	3.4	2.9
Urban (yes)	79.7	79.2	80.2	94.7	96.2	93.2	91.5	91.3	91.7
Residential segregation									
Low (z < 0)	34.9	35.4	34.5	29.0	27.0	31.0	31.1	33.4	29.5
Medium (z: 0–1.96)	43.1	43.0	43.1	36.6	36.9	36.3	30.8	30.5	31.1
High (z > 1.96)	22.0	21.5	22.3	34.4	36.1	32.7	38.1	36.1	39.5
Health behaviors									
Sleep duration									
< 7 h	31.9	31.8	32.1	31.4	30.5	32.2	41.4	40.5	42.0
7–9 h	65.0	65.3	64.6	66.8	68.2	65.4	54.3	55.3	53.6
> 9 h	3.1	2.9	3.3	1.8	1.3	2.4	4.3	4.1	4.4
Trouble falling asleep (≥ 3 times)^b^	19.7	15.5	23.5	11.5	9.5	13.4	21.0	15.9	24.7
Trouble staying asleep (≥ 3 times) ^b^	25.4	20.9	29.6	12.3	8.5	16.0	24.3	19.5	27.8
Restorative sleep (Woke up feeling rested ≥ 4 days)^b^	63.8	67.7	60.1	69.4	71.6	67.2	63.3	68.6	59.5
Sleep medication use in the past week (≥ 3 times)^b^	8.5	6.2	10.6	3.2	2.2	4.2	6.2	4.3	7.6
	Mexican Latine			Non-Mexican Latine			White (non-Hispanic)		
	All (*N* = 9,303)	Men (*n* = 4,745)	Women (*n* = 4,558)	All (*N* = 8,166)	Men (*n* = 3,812)	Women (*n* = 4,354)	All (*N* = 84,503)	Men (*n* = 40,237)	Women (*n* = 44,266)
Sociodemographic characteristics									
Age, years—mean (SE)	39.8 (0.3)	39.4 (0.3)	40.4 (0.4)	43.3 (0.2)	42.9 (0.3)	43.7 (0.3)	47.0 (0.1)	45.8 (0.2)	48.2 (0.2)
Living in poverty (<100% Federal Poverty Level)	24.0	21.2	27.0	23.7	19.0	28.2	11.2	10.2	12.1
Annual household income									
<$35,000	48.9	47.4	50.7	48.1	43.5	52.4	32.1	28.9	34.9
$35,000-$74,999	32.8	33.8	31.6	30.0	32.8	27.4	31.4	31.9	30.9
≥$75,000	18.3	18.8	17.7	21.9	23.8	20.1	36.6	39.1	34.2
Educational attainment									
< High school	35.8	39.7	31.4	22.6	23.4	21.9	7.5	8.0	7.0
High school graduate	27.6	28.2	27.0	25.2	26.5	24.0	24.8	25.9	23.8
Some college	16.3	14.7	18.2	17.4	15.9	18.8	20.1	19.7	20.5
≥ College	20.3	17.5	23.4	34.7	34.2	35.3	47.6	46.4	48.7
Unemployed/not in the labor force	24.0	17.4	31.5	30.2	23.9	36.2	33.1	27.4	38.5
Occupational category									
Professional/management	9.6	9.9	9.3	13.6	15.4	11.9	22.3	26.6	18.4
Support services	32.7	17.4	50.0	40.1	25.6	53.9	46.7	27.8	64.2
Laborers	57.7	72.7	40.7	46.2	59.0	34.1	31.0	45.6	17.4
US-born (yes)	49.8	45.0	55.1	32.6	32.2	33.0	95.5	95.3	95.6
Region of residence									
Northeast	4.8	5.6	4.0	35.0	32.7	37.2	20.3	20.2	20.5
Midwest	19.8	19.9	19.7	8.2	9.3	7.1	34.0	34.5	33.5
South	59.7	58.2	61.3	51.2	52.3	50.0	36.0	35.4	36.5
West	15.7	16.3	15.0	5.7	5.7	5.7	9.7	9.9	9.5
Housing arrangement									
Government-assisted renter	3.1	2.0	4.4	8.9	5.6	12.0	2.4	1.6	3.1
Unassisted renter	46.5	49.1	43.6	50.3	52.7	48.1	29.8	31.2	28.5
Homeowner	50.3	48.8	52.0	40.8	41.7	39.9	67.8	67.2	68.4
Housing type									
Apartment/house	90.9	89.9	91.9	96.7	96.7	96.8	95.2	95.1	95.4
Mobile home/trailer	9.1	10.1	8.1	3.3	3.3	3.2	4.8	4.9	4.6
Urban (yes)	88.0	87.4	88.7	96.0	96.0	95.9	74.7	74.2	75.2
Residential segregation									
Low (z < 0)	37.7	37.0	38.4	36.1	37.5	34.8	35.7	36.0	35.5
Medium (z: 0–1.96)	32.0	32.3	31.8	29.6	29.0	30.1	47.8	47.6	48.0
High (z > 1.96)	30.3	30.7	29.8	34.3	33.5	35.0	16.5	16.4	16.5
Health behaviors									
Sleep duration									
<7 hours	29.5	28.0	31.2	34.3	32.3	36.2	30.2	30.8	29.7
7–9 hours	67.4	68.9	65.7	63.0	65.0	61.0	66.8	66.5	67.1
>9 hours	3.1	3.0	3.1	2.8	2.7	2.8	3.0	2.8	3.2
Trouble falling asleep (≥ 3 times)^b^	17.7	13.5	22.2	19.5	14.1	24.5	20.1	16.1	23.8
Trouble staying asleep (≥ 3 times)^b^	17.9	14.8	21.3	20.7	16.4	24.8	27.4	22.8	31.7
Restorative Sleep (Woke up feeling rested ≥ 4 days)^b^	63.1	65.7	60.3	63.1	69.6	56.9	63.6	67.4	60.1
Sleep medicationuse in the past week (≥ 3 times)^b^	4.4	3.5	5.5	6.8	4.5	9.1	9.7	7.1	12.1

Data is presented as percentages or means (standard errors). All estimates are weighted for the survey’s complex sampling design. All estimates except for age are age-standardized to the US 2010 population

Abbreviations: *SE* Standard error

^a^Percentages may not sum to 100 due to missing values or rounding

^b^Trouble falling asleep, trouble staying asleep, restorative sleep, and sleep medication use data were available for 2013–2017

### Exposure Assessment: Racial and Ethnic Residential Segregation (RRS)

Consistent with prior literature, we measured the evenness domain (i.e., differential distribution of racial and ethnic groups within smaller compared to larger geographical units) and the concentration domain (i.e., hot spot/cold spot analysis identifying higher and lower concentrations of racial and ethnic groups in smaller compared to larger area) of RRS by calculating the local Getis-Ord G_i_* statistic for racial and ethnic population distribution [39–41]. NHIS participants’ home state, county, and census tract data were geographically linked to American Community Survey (ACS) 5-year estimates data for 2008–2012 (for NHIS 2011–2012 participants) and 2013–2017 (for NHIS 2013–2017 participants) [42, 43]. The ACS data included the census tract- and county-level estimated (1) total number of residents per geographical unit and the (2) total number of residents per geographical unit that identified as each racial and ethnic group. Using these estimates, we calculated racial and ethnic composition in each geographical unit (i.e., census tract and county) as the percentages of each racial and ethnic group in the unit. Subsequently, we calculated the local Getis-Ord G_i_* statistic [41], which is a z-score for each census tract that represents the degree to which the proportion of a racial or ethnic group within a census tract differs from the proportion of a racial and ethnic group in the larger area (i.e., county). A higher local Getis-Ord G_i_* statistic indicates a higher degree of RRS. Consistent with prior literature and related to the small proportion of highly integrated US neighborhoods (i.e., a low number of census tracts in the U.S. that have z-scores with negative values) [44, 45], the Getis-Ord G_i_* statistic was categorized to reflect ‘low’ (z < 0), ‘medium’ (z = 0–1.96), and ‘high’ (z > 1.96) RRS. Notably, the ‘high’ category corresponds with census tracts with values approximately two standard deviations or more (≥ 97.5th percentile) from the mean.

### Outcome Assessment: Multiple Dimensions of Sleep Health

 NHIS participants self-reported sleep duration and sleep quality by responding to questions that were similar to select items from the Pittsburgh Quality Index and Global Sleep Assessment Questionnaire [46, 47]. Participants responded to, “On average, how many hours of sleep do you get in a 24-h period?”. Responses were rounded up to the nearest hour if a participant reported a duration including ≥ 30 minutes and were rounded down to the nearest hour if a participant reported a duration including ≤ 29 minutes. Consistent with National Sleep Foundation recommendations, we categorized sleep duration as short (< 7 hours [h]), recommended (7–9 h), and long (> 9 h) [48]. Sleep quality data were collected from 2013–2017 and included frequent trouble falling asleep, trouble staying asleep, restorative sleep, and sleep medication use. For trouble falling asleep and trouble staying asleep, participants were asked, “In the past week, how many times did you have trouble [falling asleep/staying asleep]?”, and responses were dichotomized as yes (≥ 3 times/week) vs. no (< 3 times/week). For restorative sleep, participants responded to “In the past week, how many days did you wake up feeling well rested, and responses were dichotomized as yes (≥ 4 days) vs. no (< 4 days). Sleep medication use, ascertained through the question, “In the past week, how many times did you take medication to help you fall asleep or stay asleep?” was dichotomized as yes (≥ 3 times/week) vs. no (< 3 times/week).

### Potential Confounders

All potential confounders were self-reported and selected based on prior literature [5, 6, 8, 38, 49]. Sociodemographic characteristics included self-identified race and ethnicity (Asian, Black, Mexican Latine, non-Mexican Latine, White) defined using US Office of Management and Budget categories [37], binary sex/gender (men, women), age (years), annual household income (< $35,000, $35,000-$74,999, ≥ $75,000), educational attainment (< high school, high school graduate, some college, ≥ college), employment status (unemployed/not in the labor force [yes vs. no]), occupation category (professional/management, support services, laborers), and nativity status (US-born [yes vs. no]). Residence characteristics included US region of residence (Northeast, Midwest, South, West), housing arrangement (government-assisted renter, unassisted renter, homeowner), housing type (apartment/house, mobile home/trailer), urbanicity (urban vs. rural), and census tract (neighborhood)-level poverty tertiles based on the ACS 2012 and 2017 5-year estimates of the percentage of the population living below poverty in each census tract: low [0%−7.9%], medium [> 7.9%−16.9%], high [> 16.9%]. Clinical characteristics included general health status (excellent, very good, good, fair, poor).

### Potential Modifiers

Potential modifiers included self-identified race and ethnicity, sex/gender, and neighborhood-level (i.e., census tract-level) poverty.

### Statistical Analysis

All estimates were weighted to account for the complex survey design of the NHIS. We calculated age-standardized (using the 2010 US population as the standard population) descriptive statistics as means and standard deviations for continuous variables and as percentages for categorical variables. Using Poisson regression with robust variance estimation that accounts for any correlation due to the similarity of outcome (sleep) data from participants with the same census tract [50], we determined prevalence ratios (PRs) and 95% confidence intervals (CIs) for associations between ‘medium’ and ‘high’ vs. ‘low’ RRS and each sleep dimension, separately (i.e., short and long sleep duration, trouble falling asleep, trouble staying asleep, non-restorative sleep, and sleep medication use) adjusting for race and ethnicity, age (continuous), sex/gender, annual household income, educational attainment, employment status, birthplace/nativity status, US region of residence, housing arrangement, housing type, urbanicity, census tract-level poverty, and general health status. We then evaluated multiplicative interaction by race and ethnicity and sex/gender, separately, using Wald tests, and stratified results by each characteristic. Further, we evaluated intersectionality by testing for effect modification by race and ethnicity within sex/gender groups and by sex/gender within racial and ethnic groups. To assess neighborhood-level poverty as a potential effect modifier in each racial and ethnic group, we evaluated Wald tests of RRS*neighborhood-level poverty interaction terms after stratifying models by race and ethnicity. All analyses were conducted using survey procedures in SAS 9.4 or SUDAAN 11.0.3, and a two-sided *p*-value threshold of 0.05 determined statistical significance.

## Results

### Study Population Characteristics

Among 126,539 NHIS adult participants, mean age ± SE was 46 ± 0.1 years (Table 1). Overall, 15% lived below the 100% federal poverty threshold; the lowest prevalence was among White men (10%), and the highest prevalence was among Black women (30%). Most participants resided in urban counties (80%), and this prevalence was highest among non-Mexican Latine adults (96%) and lowest among White adults (75%). Overall, 35% of adults resided in neighborhoods with ‘low’ RRS, 43% lived in neighborhoods with ‘medium’ RRS, and 22% lived in neighborhoods with ‘high’ RRS. ‘High’ RRS by race and ethnicity was least prevalent among White adults (17%) followed by Mexican Latine adults (30%) as well as Asian and non-Mexican Latine adults (both 34%), while the highest prevalence of ‘high’ RRS was among Black adults (38%). Short and long sleep duration prevalence was also highest among Black adults (short sleep: 41% vs. 32% in the overall population and long sleep: 4.3% vs. 3.1% in the overall population). Prevalence of trouble falling asleep was 20% overall and highest among Black adults. Prevalence of trouble staying asleep was 25% overall and highest among White adults. Prevalence of restorative sleep was 64% with little variation by race and ethnicity except for a 69% prevalence among Asian adults. While 8.5% of all adults reported sleep medication use, the prevalence varied by race and ethnicity (range: 3.2% [Asian] – 9.7% [White]). Further, women reported consistently poorer sleep quality compared to men, both in the overall population and within racial and ethnic groups. For instance, 68% of men reported restorative sleep compared to 60% of women.

### Associations Between RRS and Multiple Dimensions of Sleep, Overall, by Race and Ethnicity, and by Sex/Gender

*Overall.* ‘Medium’ vs. low RRS was associated with a 2% lower prevalence of short sleep duration (PR = 0.98 [95% CI:0.95–1.00]) while ‘high’ vs. ‘low’ RRS was associated with a 6% lower prevalence of short sleep duration (PR = 0.94 [0.91–0.97]; Table 2). ‘High’ vs. ‘low’ RRS was associated with an 11% lower prevalence of long sleep duration (PR = 0.89 [0.80–0.99]). ‘High’ RRS was also associated with a 6% lower prevalence of trouble falling asleep (PR = 0.94 [0.90-0.98]) and a 2% higher prevalence of restorative sleep (PR = 1.02 [1.01–1.04]; Table 3). RRS was not associated with trouble staying asleep or sleep medication use in the overall population.

**Table 2:** Associations between racial and ethnic residential segregation and sleep duration, overall and stratified by race and ethnicity and sex/gender, National Health Interview Survey, 2011–2017

Racial and ethnic residential segregation	Prevalence ratio (95% confidence interval)					
	Sleep duration (reference: recommended (7–9 h), *N* = 77,317)					
	Short (< 7 h)			Long (> 9 h)		
	All (*N* = 115,559)	Men (*n* = 54,185)	Women (*n* = 61,374)	All (*N* = 80,968)	Men (*n* = 37,977)	Women (*n* = 42,991)
Overall						
Medium vs. low	0.98 (0.95–1.00)	0.98 (0.95–1.01)	0.97 (0.94–1.01)	0.97 (0.89–1.07)	1.03 (0.90–1.17)^a^	0.93 (0.83–1.04)
High vs. low	**0.94** **(0.91–0.97)**	**0.93** **(0.89–0.97)**	**0.95** **(0.91–0.99)**	**0.89** **(0.80–0.99)**	0.96 (0.82–1.12)^a^	**0.84** **(0.73–0.97)**
Asian (non-Hispanic)						
Medium vs. low	0.93 (0.83–1.05)	0.92 (0.77–1.09)	0.95 (0.81–1.11)	1.35 (0.83–2.21)	**2.81** **(1.28–6.18)**	0.92 (0.51–1.68)
High vs. low	0.96 (0.86–1.08)	0.95 (0.81–1.11)	0.97 (0.82–1.14)	1.60 (0.95–2.71)	**2.38** **(1.13–5.01)**	1.32 (0.67–2.61)
Black (non-Hispanic)						
Medium vs. low	1.00 (0.94–1.05)	0.98 (0.91–1.07)	1.00 (0.94–1.08)	1.00 (0.82–1.23)	0.91 (0.67–1.24)	1.04 (0.80–1.34)
High vs. low	0.99 (0.94–1.04)	0.98 (0.90–1.06)	1.00 (0.93–1.08)	1.02 (0.82–1.28)	1.20 (0.86–1.67)	0.92 (0.69–1.23)
Mexican Latine						
Medium vs. low	1.04 (0.95–1.14)	1.03 (0.88–1.19)	1.06 (0.94–1.20)	1.45 (1.00–2.09)^b^	**2.15** **(1.21–3.81)**	0.95 (0.57–1.59)
High vs. low	1.02 (0.93–1.13)	0.96 (0.84–1.10)	1.08 (0.94–1.25)	1.33 (0.92–1.91)^b^	1.51 (0.83–2.72)	1.24 (0.76–2.03)
Non-Mexican Hispanic/Latine						
Medium vs. low	0.99 (0.91–1.08)	1.00 (0.88–1.13)	0.99 (0.88–1.10)	1.09 (0.71–1.67)	1.31 (0.74–2.31)	0.90 (0.52–1.58)
High vs. low	0.96 (0.88–1.05)	0.93 (0.82–1.06)	0.99 (0.88–1.11)	1.24 (0.82–1.89)	1.26 (0.74–2.13)	1.25 (0.70–2.25)
White (non-Hispanic)						
Medium vs. low	0.99 (0.96–1.02)	0.99 (0.95–1.03)	0.98 (0.94–1.03)	0.93 (0.84–1.04)	0.97 (0.83–1.14)	0.91 (0.78–1.05)
High vs. low	**0.95** **(0.90–0.99)**	0.94 (0.89–1.00)	0.95 (0.90–1.01)	**0.77** **(0.65–0.91)**	0.84 (0.66–1.07)	**0.72** **(0.57–0.91)**

Models adjusted for race and ethnicity (unless stratified by race and ethnicity), sex (unless stratified by sex), age, annual household income, educational attainment, employment status, occupational category, birthplace/nativity status, US region of residence, housing arrangement, housing type, urbanicity, census tract-level poverty, and general health status

Bolded values indicate statistical significance at a two-sided *p*-value < 0.05

^a^Significant race and ethnicity*residential segregation interaction (*p* < 0.05)

^b^Significant sex/gender*residential segregation interaction (*p* < 0.05)

**Table 3:** Associations between racial and ethnic residential segregation and sleep quality, overall and stratified by race and ethnicity and sex/gender, National Health Interview Survey, 2013–2017

Racial and ethnic residential segregation	Prevalence ratio (95% confidence interval)											
	Sleep quality											
	Trouble falling asleep (≥ 3 times)			Trouble staying asleep (≥ 3 times)			Restorative sleep (Woke up rested ≥ 4 days)			Took sleep medication in the past week (≥ 3 times)		
	All (*N* = 83,504)	Men (*n* = 39,148)	Women (*n* = 44,356)	All (*N* = 83,458)	Men (*n* = 39,123)	Women (*n* = 44,335)	All (*N* = 83,265)	Men (*n* = 39,012)	Women (*n* = 44,253)	All (*N* = 83,599)	Men (*n* = 39,190)	Women (*n* = 44,409)
Overall												
Medium vs. low	0.97 (0.94–1.01)^a^	**0.92** **(0.86–0.97)**	1.01 (0.96–1.05)	0.98 (0.95–1.01)	0.98 (0.93–1.03)	0.98 (0.94–1.02)	1.00 (0.99–1.02)	1.01 (0.99–1.02)	1.00 (0.98–1.02)	1.01 (0.95–1.07)	1.02 (0.91–1.13)	1.01 (0.93–1.08)
High vs. low	**0.94** **(0.90–0.98)**^**a**^	**0.90** **(0.84–0.97)**	0.97 (0.91–1.02)	0.96 (0.92–1.00)	0.94 (0.88–1.00)	0.98 (0.93–1.03)	**1.02** **(1.01–1.04)**	1.02 (1.00–1.04)	1.03 (1.00–1.06)	1.02 (0.94–1.11)	0.96 (0.84–1.10)	1.05 (0.96–1.15)
Asian (non-Hispanic)												
Medium vs. low	0.77 (0.60–1.01)	0.67 (0.45–1.00)	0.86 (0.63–1.18)	0.86 (0.67–1.09)	0.77 (0.54–1.10)	0.93 (0.70–1.24)	1.00 (0.94–1.06)	1.00 (0.92–1.09)	1.00 (0.91–1.09)	0.74 (0.44–1.23)	0.66 (0.29–1.52)	0.80 (0.43–1.47)
High vs. low	0.91 (0.71–1.18)	0.72 (0.50–1.05)	1.10 (0.81–1.49)	0.94 (0.73–1.20)	0.86 (0.58–1.26)	1.00 (0.74–1.35)	0.96 (0.90–1.02)	0.96 (0.89–1.04)	0.96 (0.87–1.05)	1.31 (0.81–2.11)	1.65 (0.78–3.51)	1.22 (0.66–2.26)
Black (non-Hispanic)												
Medium vs. low	0.94 (0.85–1.04)	0.92 (0.75–1.11)	0.95 (0.84–1.07)	0.99 (0.90–1.08)	0.96 (0.81–1.12)	1.00 (0.90–1.12)	0.99 (0.95–1.04)	1.03 (0.97–1.10)	0.96 (0.91–1.02)	0.88 (0.70–1.10)	0.88 (0.62–1.27)	0.88 (0.67–1.15)
High vs. low	0.95 (0.85–1.06)	0.92 (0.76–1.11)	0.96 (0.85–1.10)	0.97 (0.89–1.07)	0.93 (0.79–1.10)	1.00 (0.89–1.12)	0.98 (0.94–1.03)	1.01 (0.95–1.07)	0.97 (0.91–1.03)	0.81 (0.64–1.03)	**0.64** **(0.43–0.94)**	0.89 (0.67–1.19)
Mexican Latine												
Medium vs. low	1.05 (0.90–1.23)	0.95 (0.73–1.22)	1.13 (0.93–1.36)	1.08 (0.91–1.27)	0.99 (0.74–1.32)	1.13 (0.92–1.38)	0.98 (0.92–1.04)	0.98 (0.91–1.06)	0.97 (0.90–1.06)	1.12 (0.76–1.66)	1.63 (0.89–2.98)	0.86 (0.55–1.36)
High vs. low	0.96 (0.82–1.13)	0.83 (0.63–1.09)	1.07 (0.88–1.31)	0.90 (0.75–1.09)	0.84 (0.64–1.10)	0.95 (0.74–1.21)	1.01 (0.94–1.08)	1.00 (0.92–1.08)	1.02 (0.91–1.13)	1.13 (0.76–1.67)	1.19 (0.59–2.38)	1.10 (0.67–1.80)
Non-Mexican Hispanic/Latine												
Medium vs. low	1.01 (0.86–1.18)	1.06 (0.82–1.36)	0.99 (0.82–1.19)	1.02 (0.88–1.19)	1.06 (0.81–1.38)	1.00 (0.84–1.18)	1.02 (0.96–1.09)	1.02 (0.95–1.10)	1.03 (0.93–1.13)	1.04 (0.78–1.37)	1.04 (0.66–1.64)	1.02 (0.74–1.41)
High vs. low	**0.83** **(0.72–0.97)**	**0.69** **(0.51–0.93)**	0.92 (0.77–1.10)	0.87 (0.74–1.03)	0.84 (0.64–1.10)	0.89 (0.73–1.08)	1.05 (0.99–1.11)	1.05 (0.98–1.13)	1.06 (0.96–1.16)	1.01 (0.76–1.33)	0.80 (0.50–1.27)	1.12 (0.79–1.59)
White (non-Hispanic)												
Medium vs. low	0.98 (0.94–1.02)^a^	**0.92** **(0.86–0.99)**	1.02 (0.97–1.08)	0.98 (0.94–1.01)	0.99 (0.93–1.05)	0.97 (0.93–1.01)	1.00 (0.98–1.02)	1.00 (0.97–1.02)	1.01 (0.98–1.04)	1.03 (0.96–1.10)	1.03 (0.91–1.16)	1.03 (0.94–1.12)
High vs. low	0.95 (0.89–1.01)^a^	0.94 (0.85–1.04)	0.96 (0.89–1.03)	0.97 (0.92–1.02)	0.97 (0.89–1.05)	0.98 (0.92–1.04)	**1.03** **(1.01–1.06)**	1.02 (0.98–1.05)	**1.05** **(1.01–1.08)**	1.05 (0.94–1.16)	1.01 (0.86–1.20)	1.06 (0.95–1.19)

Sleep quality measures were available for the survey years 2013–2017. Models adjusted for race and ethnicity (unless stratified by race and ethnicity), sex (unless stratified by sex), age, annual household income, educational attainment, employment status, occupational category, birthplace/nativity status, US region of residence, housing arrangement, housing type, urbanicity, census tract-level poverty, and general health status

Bolded values indicate statistical significance at a two-sided *p*-value < 0.05

^a^Sex/gender*residential segregation interaction *p* < 0.05. No race and ethnicity*residential segregation interaction terms had a *p*-value < 0.05

*By Race and Ethnicity and by Sex/Gender.* No associations between RRS and sleep duration or restorative sleep varied by race and ethnicity or sex/gender in the overall population (p-interactions > 0.05). Associations with trouble falling asleep did not vary by race and ethnicity; however, they varied by sex/gender in the overall population (p-interaction < 0.05; Table 3). Specifically, ‘medium’ vs. ‘low’ RRS was associated with an 8% lower prevalence of trouble falling asleep among men (PR = 0.92 [0.86–0.97]) but was not associated with trouble falling asleep among women (PR = 1.01 [0.96–1.05]). While estimates differed, the CIs overlapped for ‘high’ vs. ‘low’ RRS among men (PR = 0.90 [0.84–0.97]) and women (PR = 0.97 [0.91–1.02]).

### Associations Between RRS and Multiple Dimensions of Sleep by Racial and Ethnic-Sex/Gender Intersectional Group Membership

*Racial and Ethnic Differences within Sex/Gender Groups.* There were racial and ethnic differences in associations with long sleep duration among Asian and Mexican Latine men compared to White men (p_RRS*race and ethnicity_ < 0.05; Table 2). Neither ‘medium’ vs. ‘low’ (PR = 0.97 [0.83–1.14]) nor ‘high’ vs. ‘low’ (PR = 0.84 [0.66–1.07]) RRS was associated with long sleep among White men; however, ‘medium’ vs. ‘low’ RRS was associated with approximately three times the prevalence of long sleep among Asian men (PR = 2.81 [1.28–6.18]) as well as over two times the prevalence of long sleep among Mexican Latine men (PR = 2.15 [1.21–3.81]), while ‘high’ vs. ‘low’ RRS was also associated with two times the prevalence of long sleep among Asian men (PR = 2.38 [1.13–5.01]).

*Sex/gender Differences within Racial and Ethnic Groups*. There were sex/gender differences in some associations within Mexican Latine and White adults. Although ‘medium’ vs. ‘low’ RRS was associated with over two times the prevalence of long sleep among Mexican Latine men, there was no association among Mexican Latine women (PR = 0.95 [0.57–1.59]). ‘Medium’ vs. ‘low’ RRS was associated with a lower prevalence of trouble falling asleep among White men (PR = 0.92 [0.86–0.99]), but there was no association among White women (PR = 1.02 [0.97–1.08]). There were no other statistical interactions by intersectional group membership.

### Associations Between RRS and Multiple Dimensions of Sleep within Racial and Ethnic Groups by Census Tract (Neighborhood)-Level Poverty

**Fig. 1:**
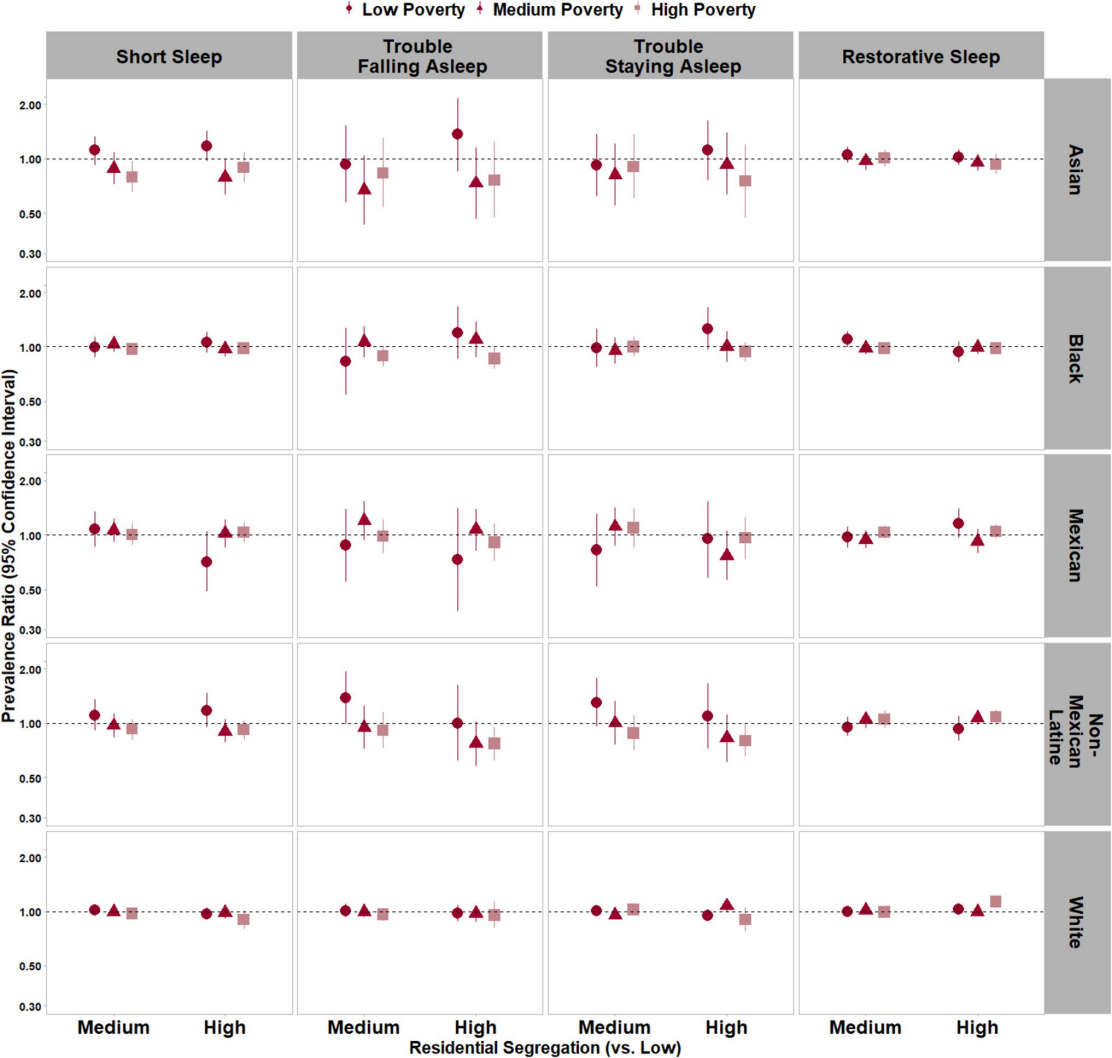
Associations of racial and ethnic residential segregation with sleep duration and sleep quality* by census tract-level poverty,** National Health Interview Survey, 2011–2017. Note: Models adjusted for sex, age, annual household income, educational attainment, employment status, occupational category, birthplace/nativity status, US region of residence, housing arrangement, housing type, urbanicity, and general health status. * Results for long sleep duration and sleep medication use are not shown but are provided in Supplemental Table 1 due to wide confidence intervals that impact scaling of data visualization. ** The census tract-level poverty*residential segregation interaction term had a *p* < 0.05 for: short sleep among Asian (non-Hispanic) adults, long sleep among Black (non-Hispanic) adults, long sleep among Mexican adults, trouble staying asleep among White adults, and restorative sleep among White adults

S*hort Sleep Duration.* Associations with short sleep varied by census tract (neighborhood)-level poverty among Asian adults (p_RRS*census tract-level poverty_ < 0.05; Fig. 1 and Supplemental Table 2). ‘Medium’ vs. ‘low’ RRS was not associated with short sleep among Asian residents of low- (PR = 1.11 [0.92–1.33]) and medium-poverty (PR = 0.88 [0.72–1.08]) neighborhoods; however, ‘medium’ RRS was associated with a 21% lower prevalence of short sleep among Asian residents of high-poverty neighborhoods (PR = 0.79 [0.65–0.97]). Further, ‘high’ RRS was marginally associated with a higher prevalence of short sleep duration (PR = 1.17 [0.97–1.42]) among Asian adults in low-poverty neighborhoods and was associated with a lower prevalence of short sleep duration among Asian adults in medium-poverty neighborhoods (PR = 0.79 [0.63–0.99]). There was no association with short sleep among NH-Asian adults in high-poverty neighborhoods (PR = 0.89 [0.74–1.08]).

*Long Sleep Duration.* Associations between ‘medium’ RRS and long sleep varied by neighborhood-level poverty among Black adults, and associations between ‘high’ RRS and long sleep varied by neighborhood-level poverty among Mexican Latine adults (p-interactions < 0.05). Specifically, Black adults in low-poverty neighborhoods with ‘medium’ vs. ‘low’ RRS had a 63% lower prevalence of long sleep duration (PR = 0.37 [0.16–0.83]); Black adults in medium-poverty neighborhoods with ‘medium’ vs. ‘low’ RRS had a marginally 34% higher prevalence of long sleep duration (PR = 1.34 [0.95–1.88]); and ‘medium’ vs. ‘low’ RRS was not associated with long sleep duration among Black adults in high-poverty neighborhoods (PR = 1.01 [0.77–1.33]). Among Mexican Latine adults, ‘high’ vs. ‘low’ RRS was associated with long sleep duration only in low-poverty neighborhoods (PR_low-poverty_ = 3.45 [1.74–6.84] vs. PR_medium-poverty_ = 0.84 [0.41–1.73] and PR_high-poverty_ = 1.28 [0.76–2.15]).

*Sleep Quality.* Data also suggested that associations between RRS and both trouble staying asleep and restorative sleep varied by neighborhood-level poverty among White adults (p-interactions < 0.05; Fig. 1). However, the CIs for associations with trouble staying asleep largely overlapped. White adults in low-poverty neighborhoods with ‘high’ vs. ‘low’ RRS had a 3% marginally higher prevalence of restorative sleep (PR = 1.03 [1.00–1.07]); White adults in medium-poverty neighborhoods with ‘high’ vs. ‘low’ RRS were no more likely to report restorative sleep (PR = 0.99 [0.95–1.03]); and White adults in high-poverty neighborhoods with ‘high’ vs. ‘low’ RRS had a 13% higher prevalence of restorative sleep (PR = 1.13 [1.06–1.20]). There were no statistically significant interactions by neighborhood-level poverty for the remaining associations within racial and ethnic groups; however, estimates often differed. For instance, among NH-Black adults, ‘medium’ vs. ‘low’ RRS was associated with a 61% lower prevalence of sleep medication use in low-poverty neighborhoods (PR = 0.39 [0.20–0.79]) but was not associated with sleep medication use in medium-poverty neighborhoods (PR = 1.18 [0.77–1.79]; Supplemental Table 2).

## Discussion

This is the first nationally representative study, to our knowledge, to empirically investigate associations between racial and ethnic residential segregation (RRS) and multiple sleep health dimensions among Asian, Black, Mexican Latine, non-Mexican Latine, and White US adults. We also sought to determine whether the associations varied by race and ethnicity, sex/gender, intersections between race and ethnicity and sex/gender, and by neighborhood-level poverty within racial and ethnic groups. RRS was generally associated with favorable sleep health, overall, which was contrary to our hypothesis. Overall, adults residing in neighborhoods with ‘medium’ and ‘high’ compared to ‘low’ levels of RRS were less likely to report short and long sleep duration. ‘Medium’ and ‘high’ RRS were also associated with a lower prevalence of trouble falling asleep, and the magnitude of association was larger among men compared to women. Moreover, adults residing in highly segregated areas had a higher prevalence of restorative sleep, and overall associations did not vary by race and ethnicity or sex/gender. Upon examination of intersectionality, there was evidence of intersectionality among men, Mexican Latine adults, and White adults. Lastly, there were inconsistent associations by neighborhood-level poverty within racial and ethnic groups that generally did not support our hypothesis of more favorable sleep health in highly segregated/lower poverty neighborhoods. Our results indicate that the potential impacts of RRS on sleep may uniquely vary by sociodemographic group and neighborhood socioeconomic status, which is plausible given the varying social conditions across groups.

Our specific results related to intersectionality and effect modification by neighborhood-level poverty were as follows: Associations with long sleep varied by race and ethnicity within men and by sex/gender among Mexican Latine adults. Further, associations with trouble falling asleep varied by sex/gender within White adults. Notable differences by neighborhood-level poverty within racial and ethnic groups occurred without consistent patterns across groups. ‘High’ segregation was associated with a higher prevalence of sleep duration outside of the recommended range among Asian and Mexican Latine adults only in low-poverty areas. Among Black adults, ‘high’ segregation was associated with a lower prevalence of long sleep duration in low-poverty areas but marginally higher prevalence of long sleep duration in medium-poverty areas. Lastly, there were stronger associations between ‘high’ segregation and higher prevalence of restorative sleep among White adults in high- versus low- and medium-poverty areas.

Few prior empirical studies have investigated RRS in relation to sleep health [27–29], which limits comparisons to prior literature. Our results may appear to be in contrast to the limited prior studies of RRS – as measured by racial and ethnic composition of census tracts and historical redlining – that showed segregation as associated with short sleep duration and non-adherence to CPAP [27–29]. Differences between our and these prior studies may explain our conflicting results. Specifically, these studies used different measures of RRS that may not sufficiently capture current segregation [40], did not include multidimensional sleep health, and had ecological designs. It is possible that associations aggregated at the population-level (ecological) in the prior studies do not reflect our observed associations at the individual-level. Further, most of the prior conceptual, non-empirical literature suggests residential segregation as an insalubrious fundamental cause that contributes to suboptimal physical and social environments among minoritized racial and ethnic groups, ultimately contributing to unfavorable sleep outcomes and sleep health disparities [8, 9, 13, 14, 25]. While segregation has been shown to concentrate negative aspects of the physical (e.g., exposure to environmental hazards) and social (e.g., poverty) environments among racially and ethnically minoritized groups [13, 14], higher concentrations of racially and ethnically minoritized groups may also produce health-promoting social environments characterized by a sense of community, strong support networks, a reprieve from interpersonal discrimination, and a shared cultural identity [21]. In contrast, minoritized groups living in less segregated neighborhoods might have access to health-promoting physical environments but face a worse social environment (e.g., increased discrimination from neighbors, lack of access to support networks, less feelings of belonging) [22, 23, 51]. Therefore, replication of the current study as well as multilevel investigations of different contexts as well as domains of RRS along with objective sleep measures are warranted.

Nonetheless, our findings of favorable sleep among adults residing in more segregated areas are consistent with prior studies demonstrating better health behaviors and mental health among minoritized racial and ethnic groups residing in segregated areas [22, 23]. Specifically, one mixed methods study among 472 Black adults aged ≥ 50 years demonstrated that living in a neighborhood with higher proportions of Black residents was associated with higher odds of meeting physical activity guidelines [22]. Qualitative interviews revealed that physical and social characteristics of the neighborhood, such as neighborhood social cohesion and safety, facilitated physical activity [22]. In another study representative of Black and White residents in Indiana (*N* = 2,685), RRS (as measured by the dissimilarity index) was associated with lower odds of major depressive disorder only among Black adults. A theorized mechanism included cohesive social networks that offer social support and other forms of social capital [23]. Notably, prior studies have also linked neighborhood social cohesion and perceived safety with favorable sleep health [9, 10, 26, 52–55]. Overall, these results suggest that community-level protective social factors may promote sleep health, especially among Black adults, thereby serving as a potential intervention target to address sleep health disparities.

More favorable sleep in highly segregated neighborhoods and the variation by race and ethnicity, sex/gender, and intersectional race and ethnicity-sex/gender groups may be explained by myriad pathways. As previously described, higher levels of social capital such as neighborhood social cohesion and safety may create more feelings of belonging in more homogenous/segregated neighborhoods – particularly among minoritized racial and ethnic groups who may otherwise face exclusion in other spaces, thus promoting better mental health and – whether real or perceived – propitious sleep health [22, 23, 52]. There was also evidence of similar associations among White adults, which would likely occur due to similar mechanisms; however, prior research largely focuses on the impacts of structural racism (including RRS) on minoritized racial and ethnic groups [13, 14, 21, 56]. Concerning the observed sex/gender differences where associations between higher segregation and trouble falling asleep were stronger among men than women in the overall population and among White participants, other studies have demonstrated that associations between other neighborhood characteristics (e.g., SES, social capital) than RRS and sleep duration as well as quality vary by sex/gender, particularly in samples predominantly comprised of White adults [57, 58]. However, it is mostly hypothesized that adverse neighborhood factors more strongly impact women than men [57]. Therefore, it is necessary to elucidate potential mechanisms among racially and ethnically diverse populations with varying immigration statuses by additionally investigating perceptions of neighborhood environments, influences of gender roles, differences in reporting of sleep complaints, and other related factors (e.g., perceived stress) in the context of residential segregation [5, 6, 57, 59]. Such research can also help explain mechanisms driving the other observations of intersectionality, including the stronger associations between higher RRS and long sleep among Mexican men compared to women as well as the higher prevalence of long sleep among Asian and Mexican Latine men in more segregated neighborhoods, which differed from the observation of no association among White men.

Variation in the RRS and sleep associations by neighborhood-level poverty within racial and ethnic groups may also be explained by various pathways and data limitations. For instance, due to the low prevalence of long sleep and relatively small sample sizes of minoritized racial and ethnic groups, estimates were imprecise. However, the observations may also be explained by differences in characteristics of the specific populations that collectively comprise low, medium, and high poverty neighborhoods. Higher segregation was associated with (1) a higher prevalence short sleep duration only in low-poverty areas among Asian adults and (2) lower prevalence of long sleep duration in low-poverty areas but marginally higher prevalence of long sleep duration in medium poverty areas among Black adults. Low-poverty neighborhoods are generally comprised of individuals of higher vs. lower relative SES. Asian and Black adults with higher versus lower occupational class (i.e., higher SES) were previously shown to have less favorable, non-recommended sleep duration [60, 61]. Psychosocial stress and other structural barriers related to occupational demands that increase with increasing professional responsibility among Asian and Black adults, in part due to racism, may contribute to fewer hours of sleep among these groups – despite their higher SES – with impacts beyond any potentially buffering (related to belongingness) by RRS [60, 61]. Further, the characteristics of segregated neighborhoods can vary by race and ethnicity even when racial and ethnic groups have similar SES. For instance, minoritized groups with higher incomes are more likely than their White counterparts to reside in neighborhoods with fewer resources that are more proximal to lower SES neighborhoods due to RRS [62]. These represent two potential pathways; however, a host of structural, environmental, and individual-level factors may further illuminate potential mechanisms. Similarly, unmeasured cultural, behavioral, and social factors may explain why ‘high’ segregation was associated with a higher prevalence of long sleep duration only in low-poverty neighborhoods among Mexican Latine adults. For instance, Mexican Latine adults concentrated in lower poverty neighborhoods may reflect enclaves in which residents have more resources and support to practice cultural/social norms related to prioritizing rest [16, 63]. Further study is warranted to explicate potential pathways underlying our observed associations.

Lastly, the stronger associations between ‘high’ segregation and restorative sleep among NH-White adults in high- versus low- and medium-poverty areas may be explained by factors related to the higher prevalence of rural residence among White adults in combination with, as previously described, feelings of belonging among a relatively homogenous group [22, 23]. Residence in rural versus suburban areas, although often related to poorer health outcomes [64], generally is related to a lower likelihood of experiencing sleep disruptors that affect sleep complaints (e.g., intermittent noise such as from traffic, sirens, etc.) [10]. Although we adjusted for urbanicity, environmental sleep disruptors that were unmeasured in this study may contribute to the higher prevalence of restorative sleep in high-poverty neighborhoods among White adults. Moreover, a recent study provided a conceptual framework describing the relationship of “whiteness” to health, positing that “whiteness” creates societal conditions that directly impact individual social characteristics and experiences (e.g., social connections, social identities, beliefs and narratives) that contribute to health [56]. It will be important to incorporate this framework to understand the role of RRS in determining sleep health among socioeconomically diverse groups of White populations.

Our findings must be considered in the context of several limitations. First, we cannot infer causal relationships using this cross-sectional study design. Additionally, the measure of RRS is limited by several factors: we lacked spatial data that better captures the spatial distribution of populations within geographical units; the use of administrative boundaries to define neighborhoods may not reflect participants’ definition of their neighborhood; and relatedly, measurement error related to the modifiable area unit problem may result in associations that may vary if neighborhood segregation was measured at another dimension than the census tract-level [65]. Moreover, we categorized the continuous segregation metric, which can result in loss of information. However, categories were meaningful in identifying varying degrees of segregation. Additionally, we captured specific domains of RRS (i.e., evenness, concentration), but it is a multidimensional measure [40]; and people may self-select into neighborhoods, which may drive associations [66]. Our sleep measures were self-reported, and prior studies have demonstrated differences in subjective and objective sleep that vary by race and ethnicity [59]. Additionally, sleep quality data were only available for some of the NHIS survey years, which may result in measurement error since some of the population was not included in that analysis. Therefore, future studies with multidimensional measures of RRS and objectively-measured sleep, longitudinal studies, and qualitative studies that capture neighborhood perceptions are needed.

Related to the study population and generalizability, the NHIS is primarily offered in English and Spanish; therefore, immigrant populations of other racial and ethnic groups (e.g., Asian) with limited English proficiency are likely underrepresented. Moreover, although we adjusted for birthplace/nativity, immigrant status may also modify associations. Further, exclusions of NHIS participants may have impacted results and led to attenuated associations since participants with higher risk profiles for poor sleep were excluded; racial and ethnic groups (e.g., American Indian/Alaska Native) were not included due to small sample size and limited power to detect associations; and most racial and ethnic groups were not disaggregated by heritage. Therefore, differences within sociodemographic groups may have been missed. Moreover, other social identities beyond the intersections of race and ethnicity and binary sex/gender were not measured (e.g., sexual and gender minoritized) but are likely important modifiers of associations.

Lastly, results should be interpreted in the context of caveats. Even if RRS is associated with better perceived sleep across groups in the current study, literature consistently demonstrates that RRS contributes to a higher likelihood of insalubrious environments and poorer physical health among minoritized racial and ethnic groups compared to White populations [13–20]. Further disaggregating groups and disentangling potential salutary and detrimental impacts of residential segregation on sleep and health is paramount. While the physical and social structures of neighborhoods across the spectrum of neighborhood-level RRS are complex, nuanced analyses may identify patterns that can be used to develop context-specific interventions to improve sleep health in different neighborhoods.

Despite these limitations, this study is the first, to our knowledge, to empirically investigate RRS in relation to multiple sleep health dimensions and capture individual-level associations among nationally representative populations of US adults. Moreover, we considered potential differences by race and ethnicity and sex/gender as well as intersectionality in associations among a large sample and included investigation of neighborhood-level poverty as a potential modifier, which is important given the hypothesized mechanisms through which RRS acts as a fundamental cause of sleep disparities [24]. Further, our study overcame limitations related to the ecological fallacy in prior literature that investigated RRS in relation to unidimensional sleep measures [27–29]. Relatedly, we overcame prior literature gaps by capturing the evenness domain of RRS, using an objective measure [40]. Additionally, we adjusted for robust set of confounders, thus reducing bias in estimated associations. Lastly, although additional research is needed to capture intragroup differences among Latine adults and across other racial and ethnic groups, we were able to estimate associations among Mexican and non-Mexican Latine groups, separately.

In conclusion, ‘high’ RRS was generally associated with more favorable self-reported sleep health among Asian, Black, Mexican Latine, non-Mexican Latine, and White US adults, which is likely explained through higher social cohesion impacting perception of neighborhood and sleep. Moreover, associations with better sleep quality were stronger among men than women and varied by neighborhood-level poverty. Further, the potentially protective associations between residential segregation and subjective short sleep were not observed among Asian and Black adults residing in low-poverty neighborhoods, likely related to structural barriers against favorable sleep health encountered by higher SES minoritized racial and ethnic groups. Among White adults, ‘high’ RRS was more strongly associated with self-reported restorative sleep in high-poverty neighborhoods. These results highlight the importance and necessity of additional empirical studies investigating RRS in relation to sleep while considering modification by sociodemographic characteristics. Future research should include investigations of mechanisms related to belonging and test neighborhood-level interventions that foster community across the spectrum of neighborhood racial and ethnic diversity while simultaneously addressing resource deprivation to combat unfavorable sleep health and sleep health disparities.

## Supplementary Information

Below is the link to the electronic supplementary material. 

ESM1 (DOCX 49.2 KB)

## Data Availability

The datasets underlying this article were derived from sources in the public domain: IPUMS Health Surveys (https://nhis.ipums.org/nhis/) and the American Community Survey (https://census.gov).
