# Supplementary material for: Racial-Ethnic Residential Segregation and Sleep Health among US Adults: Associations by Race and Ethnicity, Sex/Gender, and Neighborhood-Level Poverty
Source: J Racial Ethn Health Disparities. 2025 Mar 31;13(2):1149–64. doi: 10.1007/s40615-025-02322-y (PMC12354334; doi:10.1007/s40615-025-02322-y)
Supplement: Supplementary file 1 — (DOCX 49.2 KB) [file 40615_2025_2322_MOESM1_ESM.docx]

# Supplemental Table 1. Comparison of participants who were eligible for external data linkage to excluded participants ^a^

|  | **Participants eligible for data linkage** | **Participants excluded for not meeting inclusion criteria applied to publicly available data** |
| --- | --- | --- |
|  | **N=155,697** | **N=76,538** |
| **Sociodemographic Characteristics** |  |  |
| **Race and ethnicity ^b^** |  |  |
| Asian (non-Hispanic) | 9,260 (4.9) | 3,770 (4.1) |
| Black (non-Hispanic) | 21,583 (12.6) | 9,327 (10.9) |
| Mexican Hispanic/Latine | 16,026 (8.1) | 5,795 (5.8) |
| Non-Mexican Hispanic/Latine | 10,087 (5.5) | 3,825 (4.3) |
| White (non-Hispanic) | 98,741 (68.9) | 47,304 (67.8) |
| Other (American Indian/Alaska Native, multiracial, or other non-specified racial and ethnic group) | 0 (0.0) | 6,517 (7.0) |
| **Sex ^b^** |  |  |
| Men | 73,151 (47.8) | 30,929 (41.3) |
| Women | 82,546 (52.2) | 45,609 (58.7) |
| **Age, years – mean (SE) ^c^** | 45.8 (0.11) | 55.9 (0.14) |
| **Living in poverty (<100% Federal Poverty Level)^b^** | 24,237 (14.5) | 12,577 (17.6) |
| missing | 2,711 | 12,520 |
| **Annual household income ^b^** |  |  |
| <$35,000 | 59,424 (36.3) | 29,173 (48.2) |
| $35,000-$74,999 | 47,688 (30.6) | 15,665 (27.1) |
| ≥$75,000 | 48,585 (33.1) | 13,324 (24.7) |
| missing | 0 | 18,376 |
| **Educational attainment ^b^** |  |  |
| <High school | 19,843 (11.3) | 13,539 (16.5) |
| High school graduate | 38,316 (24.2) | 20,456 (26.9) |
| Some college | 31,075 (20.1) | 14,579 (19.3) |
| ≥ College | 66,463 (44.5) | 26,955 (37.3) |
| missing | 0 | 1,009 |
| **Unemployed/not in the labor force ^b^** | 49,542 (32.4) | 48,293 (63.9) |
| missing | 0 | 131 |
| **US-born** (yes) ^b^ | 126,790 (83.6) | 64,100 (85.5) |
| missing | 0 | 191 |
| **Region of residence ^b^** |  |  |
| Northeast | 25,432 (17.5) | 12,725 (17.9) |
| Midwest | 34,280 (24.0) | 15,946 (22.7) |
| South | 55,423 (36.5) | 27,679 (37.4) |
| West | 40,562 (22.0) | 20,188 (22.1) |
| **Housing arrangement ^b^** |  |  |
| Government-assisted renter | 7,068 (4.0) | 5,149 (6.1) |
| Unassisted renter | 57,613 (36.1) | 22,598 (28.2) |
| Homeowner | 91,016 (59.9) | 48,079 (65.7) |
| missing | 0 | 712 |
| **Housing type ^b^** |  |  |
| Apartment/house | 148,388 (95.6) | 71,999 (95.0) |
| Mobile home/trailer | 7,309 (4.4) | 3,988 (5.0) |
| missing | 0 | 551 |
| **Health Behaviors ^b^** |  |  |
| Sleep duration |  |  |
| <7 hours | 48,588 (31.7) | 22,432 (30.1) |
| 7-9 hours | 99,261 (65.2) | 46,642 (63.8) |
| ≥9 hours | 4,742 (3.1) | 4,630 (6.2) |
| missing | 3,106 | 2,834 |
| Trouble falling asleep (≥3 times) ^b^ | 21,276 (19.7) | 12,458 (22.7) |
| missing | 49,545 | 23,000 |
| Trouble staying asleep (≥3 times) ^b^ | 27,327 (25.3) | 16,856 (31.0) |
| missing | 49,599 | 23,036 |
| Restorative sleep  (Woke up feeling rested ≥4 days/week) ^b^ | 67,075 (63.8) | 34,083 (64.7) |
| missing | 49,857 | 23,303 |
| Sleep medication use in the past week  (≥3 times) ^b^ | 8,989 (8.4) | 6,663 (12.4) |
| missing | 49,419 | 22,815 |
| **Clinical Characteristics** |  |  |
| General health status ^b^ |  |  |
| Excellent | 45,099 (29.7) | 14,835 (19.5) |
| Very good | 53,128 (34.6) | 20,817 (27.9) |
| Good | 40,588 (25.4) | 23,099 (30.2) |
| Fair | 13,687 (8.3) | 12,738 (16.3) |
| Poor | 3,195 (1.9) | 4,945 (6.2) |
| missing | 0 | 104 |

Abbreviations: SE (standard error)

Note: Data are presented as column percentages or means (standard errors). All estimates are weighted for the survey’s complex sampling design. All estimates except for age are age-standardized to the US 2010 population.

^a^ Percentages may not sum to 100 due to missing values or rounding. Trouble falling asleep, trouble staying asleep, restorative sleep, and sleep medication use data were available for 2013-2017.

^b^ Chi-square p<0.05.

^c^ T-test p<0.05.

**Supplemental Table 2. Associations of racial and ethnic residential segregation with sleep duration and sleep quality by census-tract level poverty, National Health Interview Survey, 2011-2017**

| **Racial and Ethnic Residential Separation** | **Prevalence Ratio (95% Confidence Interval)** | | | | | | | | | | | | | | | | | |
| --- | --- | --- | --- | --- | --- | --- | --- | --- | --- | --- | --- | --- | --- | --- | --- | --- | --- | --- |
|  | **Sleep Quality ^a^** | | | | | | | | | | | | **Sleep Duration**  **(reference: recommended (7-9 hours))** | | | | | |
|  | **Trouble Falling Asleep**  **(≥3 times)**  **n=16,660 vs. n=66,844** | | | **Trouble Staying Asleep**  **(≥3 times)**  **n=21,638 vs. n=61,820** | | | **Restorative Sleep**  **(Woke up rested**  **≥4 days)**  **n=52,762 vs. n=30,503** | | | **Took sleep medication in the Past Week**  **(≥3 times)**  **n=7,171 vs. n=76,428** | | | **<7 hours**  **n=38,242 vs. n=77,317** | | | **>9 hours**  **n=3,651 vs. n=77,317** | | |
| **Neighborhood (census tract-level) poverty** | **Low n=26,153** | **Medium**  **n=28,497** | **High**  **n=28,854** | **Low n=26,135** | **Medium**  **n=28,483** | **High**  **n=28,840** | **Low**  **n=26,093** | **Medium**  **n=28,421** | **High**  **n=28,751** | **Low n=26,175** | **Medium**  **n=28,522** | **High**  **n=28,902** | **Low n=35,902** | **Medium**  **n=38,911** | **High**  **n=40,746** | **Low**  **n=25,910** | **Medium**  **n=27,103** | **High**  **n=27,955** |
| **Asian**  **(non-Hispanic)** |  |  |  |  |  |  |  |  |  |  |  |  |  |  |  |  |  |  |
| Medium vs. Low | 0.93  (0.57-1.53) | 0.67  (0.43-1.04) | 0.83  (0.54-1.30) | 0.92  (0.62-1.36) | 0.81  (0.55-1.21) | 0.90  (0.60-1.37) | 1.05  (0.95-1.16) | 0.97  (0.87-1.07) | 1.01  (0.90-1.12) | 0.90  (0.37-2.16) | 0.63  (0.26-1.53) | 0.53  (0.24-1.17) | 1.11  (0.92-1.33)^b^ | 0.88  (0.72-1.08)^b^ | **0.79**  **(0.65-0.97)^b^** | 1.31  (0.64-2.70) | 0.76  (0.34-1.71) | 1.88  (0.61-5.79) |
| High vs.  Low | 1.36  (0.85-2.16) | 0.73  (0.46-1.15) | 0.76  (0.47-1.23) | 1.11  (0.76-1.63) | 0.93  (0.63-1.39) | 0.75  (0.47-1.20) | 1.02  (0.92-1.13) | 0.95  (0.86-1.06) | 0.93  (0.82-1.06) | 1.71  (0.76-3.83) | 1.71  (0.69-4.23) | 0.54  (0.22-1.33) | 1.17  (0.97-1.42)^b^ | **0.79**  **(0.63-0.99)^b^** | 0.89  (0.74-1.08)^b^ | 1.61  (0.73-3.55) | 1.71  (0.72-4.04) | 1.19  (0.36-3.89) |
| **Black**  **(non-Hispanic)** |  |  |  |  |  |  |  |  |  |  |  |  |  |  |  |  |  |  |
| Medium vs. Low | 0.83  (0.54-1.27) | 1.06  (0.87-1.29) | 0.89  (0.78-1.01) | 0.98  (0.77-1.26) | 0.95  (0.80-1.12) | 1.00  (0.88-1.13) | 1.10  (0.99-1.22) | 0.98  (0.91-1.05) | 0.98  (0.91-1.04) | **0.39**  **(0.20-0.79)** | 1.18  (0.77-1.79) | 0.85  (0.65-1.12) | 0.99  (0.87-1.14) | 1.03  (0.94-1.12) | 0.97  (0.91-1.04) | **0.37**  **(0.16-0.83)^b^** | 1.34  (0.95-1.88)^b^ | 1.01  (0.77-1.33)^b^ |
| High vs.  Low | 1.19  (0.85-1.67) | 1.10  (0.87-1.38) | **0.86**  **(0.76-0.99)** | 1.26  (0.96-1.65) | 1.00  (0.82-1.22) | 0.94  (0.83-1.06) | 0.94  (0.82-1.07) | 0.99  (0.91-1.07) | 0.98  (0.92-1.04) | 0.55  (0.23-1.30) | 0.86  (0.54-1.36) | 0.78  (0.59-1.04) | 1.06  (0.93-1.21) | 0.97  (0.88-1.08) | 0.98  (0.91-1.05) | 0.72  (0.36-1.46)^b^ | 0.87  (0.52-1.46)^b^ | 1.13  (0.87-1.46)^b^ |
| **Mexican Latine** |  |  |  |  |  |  |  |  |  |  |  |  |  |  |  |  |  |  |
| Medium vs. Low | 0.88  (0.55-1.39) | 1.20  (0.93-1.54) | 0.98  (0.79-1.22) | 0.83  (0.52-1.31) | 1.11  (0.87-1.42) | 1.09  (0.85-1.40) | 0.97  (0.85-1.11) | 0.94  (0.84-1.05) | 1.03  (0.95-1.12) | 0.92  (0.43-0.98) | 1.36  (0.81-2.29) | 0.75  (0.46-1.21) | 1.08  (0.86-1.35) | 1.06  (0.91-1.23) | 1.00  (0.88-1.15) | 1.04  (0.38-2.86)^b^ | 1.24  (0.74-2.10)^b^ | 1.50  (0.85-2.65)^b^ |
| High vs.  Low | 0.73  (0.38-1.40) | 1.07  (0.82-1.38) | 0.91  (0.72-1.15) | 0.95  (0.58-1.54) | 0.77  (0.56-1.05) | 0.96  (0.73-1.25) | 1.16  (0.96-1.40) | 0.92  (0.79-1.07) | 1.04  (0.95-1.14) | 2.37  (0.98-5.76) | 0.57  (0.24-1.33) | 0.97  (0.60-1.57) | 0.71  (0.49-1.04) | 1.02  (0.85-1.22) | 1.03  (0.91-1.17) | **3.45**  **(1.74-6.84)^b^** | 0.84  (0.41-1.73)^b^ | 1.28  (0.76-2.15)^b^ |
| **Non-Mexican Hispanic/Latine** |  |  |  |  |  |  |  |  |  |  |  |  |  |  |  |  |  |  |
| Medium vs. Low | 1.38  (0.99-1.92) | 0.95  (0.72-1.25) | 0.91  (0.73-1.15) | 1.30  (0.96-1.77) | 1.00  (0.76-1.33) | 0.88  (0.71-1.10) | 0.95  (0.85-1.08) | 1.05  (0.94-1.16) | 1.05  (0.94-1.17) | 1.16  (0.68-1.98) | 1.25  (0.77-2.03) | 0.87  (0.59-1.28) | 1.11  (0.91-1.35) | 0.97  (0.83-1.13) | 0.93  (0.81-1.05) | 1.17  (0.56-2.46) | 1.48  (0.65-3.37) | 0.91  (0.55-1.51) |
| High vs.  Low | 1.00  (0.62-1.62) | 0.77  (0.58-1.02) | 0.77  (0.62-0.95) | 1.09  (0.72-1.66) | 0.83  (0.61-1.12) | **0.80**  **(0.65-0.99)** | 0.93  (0.80-1.09) | 1.07  (0.98-1.18) | 1.08  (0.98-1.19) | 1.34  (0.72-2.52) | 0.85  (0.52-1.40) | 0.96  (0.67-1.37) | 1.17  (0.95-1.46) | 0.90  (0.78-1.05) | 0.92  (0.81-1.03) | 1.30  (0.47-3.60) | 2.69  (1.35-5.36) | 0.79  (0.49-1.27) |
| **White**  **(non-Hispanic)** |  |  |  |  |  |  |  |  |  |  |  |  |  |  |  |  |  |  |
| Medium vs. Low | 1.01  (0.93-1.10) | 0.99  (0.93-1.06) | 0.96  (0.88-1.03) | 1.01  (0.94-1.08)^b^ | 0.95  (0.90-1.01)^b^ | 1.02  (0.95-1.08)^b^ | 1.00  (0.97-1.03)^b^ | 1.01  (0.98-1.04)^b^ | 0.99  (0.95-1.02)^b^ | 0.97  (0.85-1.10) | 1.11  (0.99-1.24) | 0.98  (0.87-1.11) | 1.02  (0.97-1.08) | 0.99  (0.95-1.03) | 0.97  (0.93-1.02) | 0.96  (0.77-1.21) | 0.98  (0.83-1.17) | 0.92  (0.78-1.10) |
| High vs.  Low | 0.98  (0.88-1.08) | 0.97  (0.87-1.08) | 0.95  (0.81-1.13) | 0.95  (0.88-1.03)^b^ | 1.07  (0.99-1.17)^b^ | 0.90  (0.77-1.05)^b^ | 1.03  (1.00-1.07)^b^ | 0.99  (0.95-1.03)^b^ | **1.13**  **(1.06-1.20)^b^** | 0.94  (0.80-1.10) | 1.12  (0.96-1.31) | 1.29  (0.99-1.68) | 0.97  (0.91-1.05) | 0.98  (0.91-1.05) | 0.90  (0.80-1.01) | 0.78  (0.59-1.01) | 0.93  (0.71-1.22) | 0.53  (0.30-0.93) |

^a^ Sleep quality was available for survey years 2013-2017.

^b^ Census-tract level poverty*residential separation interaction p<0.05.

Note: Models adjusted for sex (unless stratified by sex), age, annual household income, educational attainment, employment status, occupational category, birthplace/nativity status, US region of residence, housing arrangement, housing type, urbanicity, and general health status.

Bolded values indicate statistical significance at a two-sided p-value <0.05.
